# Set1 is a critical transcriptional regulator in response to external signals in *Candida albicans*

**DOI:** 10.1093/nar/gkaf632

**Published:** 2025-07-08

**Authors:** Jueun Kim, Jiyeon Park, Eun-Jin Lee, Yong-Joon Cho, Jung-Shin Lee

**Affiliations:** Department of Molecular Bioscience, College of Biomedical Science, Kangwon National University, Chuncheon 24341, Republic of Korea; Institute of Life Sciences, Kangwon National University, Chuncheon 24341, Republic of Korea; Department of Molecular Bioscience, College of Biomedical Science, Kangwon National University, Chuncheon 24341, Republic of Korea; Department of Life Sciences, Korea University, Seoul 02841, Republic of Korea; Department of Molecular Bioscience, College of Biomedical Science, Kangwon National University, Chuncheon 24341, Republic of Korea; Multidimensional Genomics Research Center, Kangwon National University, Chuncheon 24341, Republic of Korea; Department of Molecular Bioscience, College of Biomedical Science, Kangwon National University, Chuncheon 24341, Republic of Korea

## Abstract

Precise gene regulation is vital for maintaining cellular homeostasis and enabling environmental adaptation in living organisms. Eukaryotic organisms display intricate transcriptional regulation influenced by chromatin structures and histone modifications. Set1-mediated methylation of histone H3 at lysine 4 (H3K4) is a well-established marker of active transcription in eukaryotes. However, the deletion of Set1 in the dimorphic pathogenic fungus, *Candida albicans*, led to the activation of signal-responsive genes even in the absence of external signals. The present study aimed to elucidate Set1’s role in the transcription of genes that respond to external signals. These inducible genes displayed atypical H3K4 modification patterns. During the initial induction stages, H3K4 methylation was not involved in the rapid and robust expression of these genes; instead, acetylation of the same residue was involved. H3K4 acetylation significantly increased in the absence of H3K4 methylation, allowing genes that did not receive external transcriptional signals to initiate mRNA expression, leading to morphological changes. With continued exposure to induction signals, the heightened H3K4 acetylation decreased while H3K4 methylation increased in these genes. Thus, inducible genes receive positive feedback for stable and sustainable expression. In conclusion, Set1 precisely modulates H3K4 methylation and acetylation levels to regulate the transcription of inducible genes at specific times and levels.

## Graphical Abstract

**Graphical Abstract:**
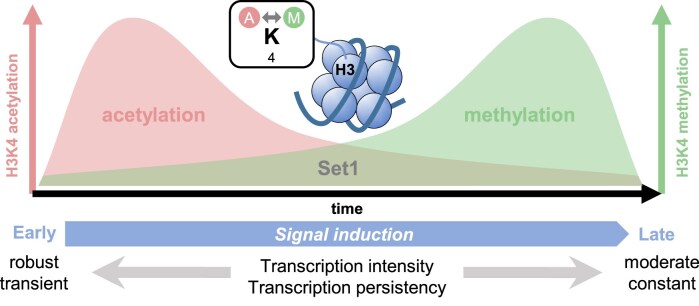

## Introduction

Genes in all living organisms, from bacteria to humans, must be tightly regulated to ensure their proper expression. Most genes are not differentially expressed and maintain their expression at the appropriate levels because their fundamental cellular functions remain the same regardless of environmental conditions. However, for life to adapt to the environment, it is necessary to control the expression levels of specific genes according to environmental changes. The expression of genes that are influenced by the environment is tightly regulated, particularly through signal transduction pathways that enable gene expression in response to specific signals [1–5]. Aberrant expression of these genes in the absence of appropriate signals can lead to various diseases, including cancer [6].

In eukaryotic organisms, both transcriptional activation and repression are directly influenced by specific chromatin structures, including posttranslational histone modifications [4, 7]. These chemical modifications of histone residues regulate the chromatin structure by collaborating with their modifying enzymes or other chromatin-modifying factors, such as nucleosome remodeling factors, thus allowing transcription factors to act properly [8–11]. Among these, histone methylation can have opposite effects on transcription, depending on the methylated residue [12]. For example, methylation of histone H3 at lysine 4 (H3K4) is involved in active transcription. H3K4 methylation is a well-studied histone modification owing to its positive role in transcription [13, 14]. Each of the three states of H3K4 methylation has a distinct localization pattern in genes; among these, the H3K4 tri-methylation (H3K4me3) is recognized as a marker of active transcription because of its abundance at the 5′ end, which contains transcriptional start sites (TSSs) of actively transcribed genes [13–16]. Also, it is well established that the broad H3K4me3 domain plays a crucial role in transcriptional activities associated with cell identity [17–20] and H3K4me3 facilitates RNA polymerase II pause-release and elongation [21].

Although the positioning of H3K4me3 on actively transcribed genes in a steady state is well documented, the direct role of H3K4me3 in the transcriptional activation of inactive genes remains unclear. In other words, given that the loss of H3K4me3 has minimal effects on global transcription in many model organisms, there has been significant debate regarding whether H3K4me3 actually activates gene expression [22]. In addition to methylation, H3K4 can also be modified with an acetyl group, although H3K4 acetylation has not been extensively studied. Indeed, it is reported that H3K4 acetylation is associated with active transcription and is typically found at the TSS of genes [14, 23]. Thus, both H3K4me3 and H3K4 acetylation are linked to active gene expression and are primarily localized near the TSS. However, the precise effect of this interplay on gene expression remains poorly understood.

The most common fungal pathogen, *Candida albicans*, is a good model for studying transcriptional regulation mechanisms. It is a member of the normal flora found on the skin and mucosal surfaces of most healthy individuals; however, it can transform into an opportunistic pathogen in response to environmental changes in the host [24, 25]. *C. albicans*, which exists as non-pathogenic normal flora, alters the expression of various virulence-related genes in response to external factors, that is, the host environment leading to its transition from a non-virulent commensal to a virulent pathogen [26–28]. One outstanding feature of *C. albicans* in terms of its virulence is its ability to exist in various morphological forms, from unicellular yeast form to filamentous form, including pseudohyphae and true hypha [29]. *C. albicans* activates the expression of various genes necessary for hyphal formation [30]. One of the features of hyphal formation in *C. albicans* is the transcriptional induction of hypha-specific genes in response to external environmental signals, resulting in the synthesis of cell wall proteins found in *C. albicans* filaments, which play a crucial role in adhesion to host epithelial cells [31]. These genes are generally repressed under normal conditions; however, their transcription is dramatically induced when *C. albicans* is exposed to conditions that induce hyphal growth [32–35].

We previously observed that the overall gene expression in *C. albicans* did not significantly change in the absence of the H3K4 methyltransferase Set1 [36]. These findings were consistent with those of previous studies conducted by other researchers [37–42]. Interestingly, the number of genes with upregulated expression was higher than the number of genes with decreased expression in the absence of Set1 and Set1-mediated H3K4 methylation. Nevertheless, the high enrichment of H3K4 methylation in actively expressed genes underscores the significance of H3K4 methylation in gene expression and it cannot be overlooked. Thus, we aimed to elucidate the role of Set1 in genes that exhibited increased expression in the absence of Set1 and Set1-mediated H3K4 methylation. In the present study, we confirmed that Set1 precisely regulates the expression of genes induced by specific environmental signals. Furthermore, we revealed that Set1 plays a key regulatory role in directly controlling the expression of genes induced by environmental cues by balancing methylation and acetylation of the same H3K4 residue.

## Materials and methods

### Strains and media

The *set1* defective strain (referred to as *dset1* in this study), which contains an internal deletion of the RRM domain but retains the SET domain, was given by Dr Clancy’s group [43] and the *gcn5* deleted strain was provided by Dr Kuchler’s group [44]. The *set1* knockout strain (Δ*set1*), in which the entire *SET1* open reading frame was deleted, was generated using the CRISPR–Cas9 system adapted from [45]. A schematic overview of these Set1 mutant alleles and their domain structures is shown in Supplementary Fig. S1. *C. albicans* cells were grown in YPD media (1% yeast extracts, 2% peptone, 2% glucose) at 30°C. For inducing hyphal growth, precultured cells in YPD were inoculated into YPD supplemented with 10% fetal bovine serum (FBS; Gibco, 26140-079) and incubated at 37°C. The clinical isolate SC5314 was used as the wild-type (WT) strain in this study.

### Preparation of nuclear extract and western blotting

Overnight cultured cells were lysed using a nuclear isolating buffer (250 mM sucrose, 60 mM KCl, 14 mM NaCl, 50 mM MgCl_2_, 10 mM CaCl_2_, and 0.8% Triton X-100) with 0.5-mm glass beads. After centrifugation at 3000 rpm for 5 min to separate the lysate, the pellets were boiled with SDS loading buffer for 5 min. Nuclear extracts were subsequently used for western blotting and probed with the following antibodies: anti-H3, anti-H3K4me1, anti-H3K4me2, and anti-H3K4me3 (a kind gift provided by Dr A. Shilatifard, Northwestern University, Chicago, USA), and anti-H3K4ac (Abcam, ab113672). 1:10 000 goat anti-rabbit IgG (Enzo, ADI-SAB-300-J) was used for secondary antibody.

### RNA isolation and sequencing analysis

Total RNA was extracted from each sample using the NucleoSpin^®^ RNA Kit (MN, MN740955) according to the protocol of the manufacturer. *C. albicans* cells were grown in YPD and harvested during the exponential growth phase, at an OD_600_ of 1.0 for yeast conditions. To prepare hyphal status cells, *C. albicans* grown in YPD were harvested at OD_600_ of 0.9 and transferred to preheated YPD containing 10% FBS. Hyphal formation was induced by incubating cells at 37°C with shaking. For sequencing, mRNA was captured using the NEBNext^®^ Poly(A) mRNA Magnetic Isolation Module (NEB, E7490), and a strand-specific sequencing library was synthesized using the NEBNext^®^ Ultra™ Directional RNA Library Prep Kit for Illumina (NEB, E7420), according to the instruction manual. The raw sequencing reads were trimmed using Trimmomatic [46]. Subsequently, HISAT2 and SAMtools were employed for mapping to the reference genome, followed by sorting [47, 48]. Mapped reads were counted using featureCounts [49]. To compare the differential expression between WT and *dset1*, we applied DEseq2 normalization [50]. The expression data were visualized using a heatmap generated with the Pheatmap R package and the Integrative Genomics Viewer (IGV) genome browser track [51]. In addition, Cluster 3.0 [52] and Java TreeView [53] were used for cluster and visualization. Gene Ontology (GO) analysis was performed using two different tools, depending on the GO category. For biological process analysis, the GO Slim Mapper tool from the *Candida* Genome Database, which provides a structured classification of genes based on their most frequently associated GO terms, was used to classify genes into broader functional categories. For cellular compartment analysis, we used the DAVID bioinformatics tools, which enables functional grouping of genes into enriched GO categories. RNA-seq experiments were performed in triplicate under hyphal conditions to ensure accurate classification of gene induction, while yeast condition samples were analyzed in duplicate.

### Quantitative reverse transcription PCR

Total RNA was extracted using the NucleoSpin^®^ RNA Kit (MN, MN740955) according to the manufacturer’s protocol. The concentration and purity of the RNA were assessed using a NanoDrop spectrophotometer (Thermo Fisher Scientific), ensuring an *A*_260_/*A*_280_ ratio of 1.8–2.0. Complementary DNAs (cDNAs) were synthesized from the total RNA using the PrimeScript™ 1st Strand cDNA Synthesis Kit (Takara, 6110A) according to the manufacturer’s instructions. Quantitative reverse transcription PCR (qRT-PCR) was performed in a 20 µl reaction mixture containing 5 µl cDNA (100 ng), 5 µl primers (final concentration 0.2 µM each), and 10 µl SYBR Green PCR Master Mix (Toyobo, TOQPS-201). Relative gene expression levels were calculated using the ΔΔCt method, with *EFB1* as the internal control. Each reaction was performed in technical triplicates and at least three biological replicates were used for each condition. Primers used for qRT-PCR are listed in Supplementary Table S1.

### Chromatin immunoprecipitation and sequencing analysis

The chromatin immunoprecipitation (ChIP) assay was conducted following the protocol previously described [54]. Cells were grown until they reached an OD_600_ of 1.0 and then fixed to crosslink the proteins to the DNA using 1% formaldehyde, followed by quenching with 125 mM glycine. To induce hyphal formation in *C. albicans* cells, they were harvested at an OD_600_ of 0.9 and transferred to preheated YPD supplemented with 10% FBS. Hyphal growth was prompted by incubating the cells at 37°C with shaking. After sonication, chromatin extracts were then incubated with antibodies for 3 h at 4°C. The antibodies used in the ChIP assay included anti-H3, anti-H3K4me1, anti-H3K4me2, anti-H3K4me3, and anti-H3K4ac. The ChIP DNA samples were subsequently analyzed by quantitative PCR (qPCR) using the SYBR Green PCR mix (Toyobo, TOQPS-201) and the Applied Biosystems 7500 Real-Time PCR System. The ChIP-qPCR data were analyzed using percent input normalization, where Ct values of ChIP-enriched DNA were normalized against input DNA. The primer sequences used in the qRT-PCR assay are listed in Supplementary Table S1. For ChIP-seq, the sequencing libraries were prepared using the NEBNext^®^ ChIP-Seq Library Prep Master Mix Set for Illumina (NEB, E6240) following the manufacturer’s instructions. The trimmed ChIP-seq reads were mapped using Bowtie 2 [55]. For visualization, EaSeq and IGV genome browser [51, 56] were used. ChIP-seq data were generated from biological duplicates for each condition. For analysis between samples in consideration of biological replicates, raw sequencing reads between replicates were combined and used for further processing. For spike-in normalization, 5% *Schizosaccharomyces pombe* chromatin was added to each sample as an internal reference, and the number of reads mapped to *S. pombe* chromatin was adjusted to 500 000 reads. The ratio of mapped reads for each sample was then normalized based on this adjustment, accounting for differences in sequencing depth and accurately reflecting the relative changes in histone modifications.

### Statistical analysis of data reproducibility

To evaluate the reproducibility of RNA-seq and ChIP-seq datasets, we calculated Pearson correlation coefficients and performed hierarchical clustering. Log(DESeq2-normalized counts) between biological replicates were compared to assess correlation (Supplementary Fig. S10). To account for batch effects, we used the sum of biological replicates for downstream analysis.

## Results

### H3K4 methyltransferase deletion results in enhanced expression of certain genes

**Figure 1.**
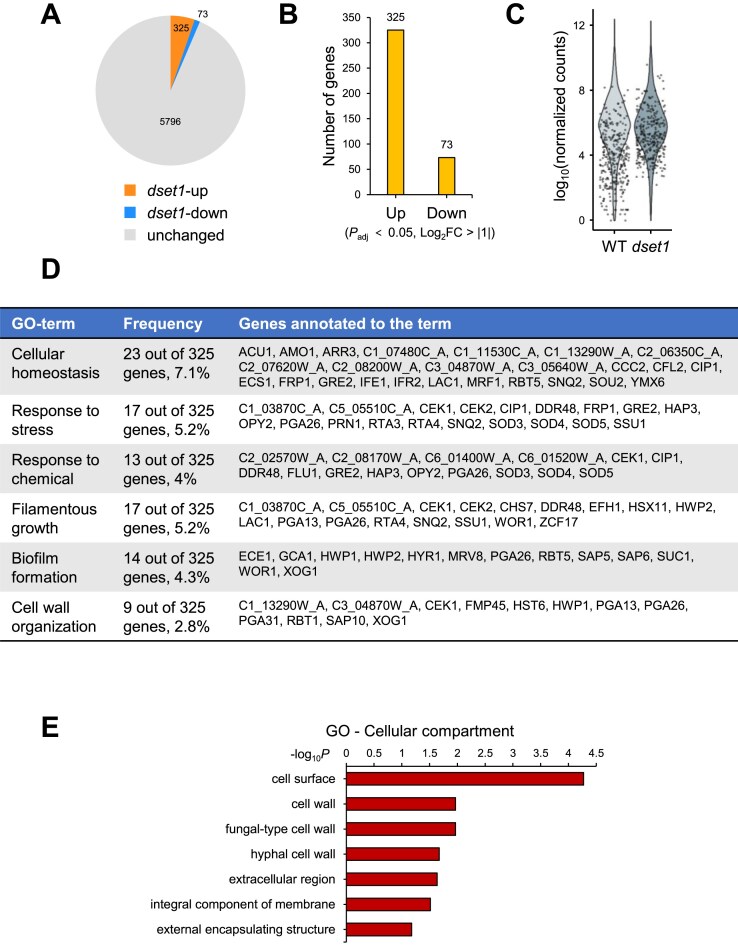
Deletion of Set1 leads to increased expression of select inducible genes. (A, B) In the absence of Set1, there were genes that showed enhanced expression. The numbers of up- and downregulated genes [*P*_adj_ < .05, log_2_ fold change (FC) > 1] in the *dset1* strain compared to the WT were represented as a pie chart in panel (**A**) and a bar plot in panel (**B**). The number of upregulated genes are more than four times higher than that of the downregulated genes in the Set1 deletion mutant. (**C**) The basal expression levels of most *dset1*-upregulated genes are remarkably low. In other words, the genes that exhibit increased expression in *dset1* are genes that should not be expressed in WT. The overall gene expression was illustrated as a violin plot, and the 325 *dset1*-upregulated genes are highlighted with dots. (D, E) The *dset1*-upregulated genes are typically subject to repression under normal conditions and are activated only in response to specific signals. GO analysis was conducted and is presented for biological processes in panel (**D**) and cellular compartments in panel (**E**).

H3K4 methylation is a well-known active transcription marker because its peak levels correlate with gene expression levels. Thus, it can be speculated that in the absence of H3K4 methylation, gene expression does not occur. However, in several research models, the absence of H3K4 methylation did not significantly alter genome-wide transcription, with changes observed in the expression levels observed of a subset of genes [22, 36, 42]. If H3K4 methylation is merely a consequence of gene expression and does not directly contribute to transcriptional regulation, its absence should not result in any changes in gene expression. Interestingly, when comparing the expression levels between the WT and the H3K4 methyltransferase Set1 defective strain (*dset1*) using *C. albicans* as a research model, we observed that the number of genes with more than a two-fold increase in expression (325 genes) was over four times greater than the number of genes with more than a two-fold decrease in expression (75 genes; Fig. 1A and B). Surprisingly, we observed that most genes with increased expression in *dset1* exhibited low basal expression levels in WT (Fig. 1C). In other words, the regulation of genes that should not be expressed was disrupted; thus, these genes were abnormally expressed in the absence of Set1. GO enrichment analysis revealed that the genes showing increased expression in *dset1* were involved in biological processes, such as stress response, chemical response, and hypha or biofilm formation, which are negatively regulated under normal conditions and induced only under specific environmental conditions (Fig. 1D). In addition, most of the *dset1*-increased gene products were localized on the cell surface and cell wall (Fig. 1E).

### Inducible genes that influence the phenotype were expressed in the absence of Set1

Hypha-specific genes are expressed only in specific environments and result in the synthesis of proteins that are localized in the cell wall of *C. albicans*. These genes are repressed under normal conditions and are expressed only when *C. albicans* receives hypha-inducing signals. The deletion of Set1 reportedly induced a more hyper-filamentous form in the embedded growth of *C. albicans* on YPD reverse agar [43]. Therefore, we investigated whether hypha-specific genes were expressed upon deletion of Set1, even in the absence of hypha-inducing signals.

**Figure 2.**
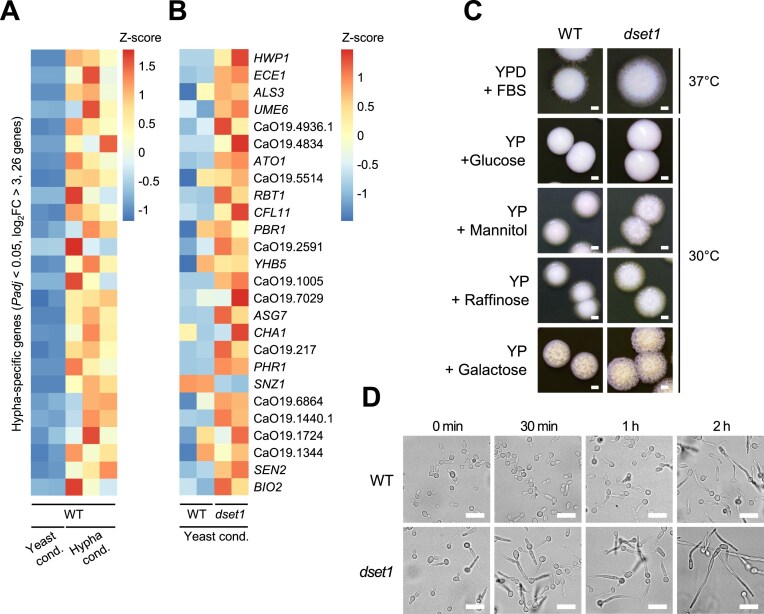
The expression of hypha-specific genes is enhanced in the absence of Set1. (**A**-**B**) Set1 deletion causes derepression of hypha-specific genes. Raw RNA-seq data were normalized by library size using DESeq2. The top 32 genes, exhibiting more than eight-fold change in expression under hyphal condition compared to the normal condition in WT, are displayed. The majority of 32 hypha-specific genes showed upregulation in the *dset1* relative to WT, even under yeast condition. *Z*-scores were calculated for each plot and visualized using the Pheatmap R package. (**C**) *dset1* are hyper-filamentous in serum-induced condition as well as various carbon source media. Overnight cultured *C. albicans* was diluted and spread on agar plates with the indicated carbon sources or 10% FBS. The plates were then incubated at the indicated temperatures for 5 days and colony morphology was observed under a stereoscopic microscope (the scale bar means 1 mm). (**D**) *dset1* forms hypha rapidly relative to WT. Cells were induced to form hypha at 37°C in liquid media containing 0.1% glucose and 10% FBS. At the indicated time points, cell supernatants were harvested and observed under a microscope (the scale bar means 10 μm).

Although Set1-mediated H3K4 methylation is a well-known active transcription marker, we observed increased expression of hypha-inducing genes in the *dset1* strain, and the levels of hypha-repressed genes in this strain were lower than those in WT (Supplementary Fig. S2A and B). Particularly, we found that hypha-specific genes, which were upregulated more than eight-fold in hyphal form [*n* = 26, log_2_FC > 3, adjusted *P*-value (*P*_adj_) <.05], were expressed in yeast form of *dset1* (Fig. 2A and B). Of these upregulated genes, the expression levels of *HWP1*, *ALS3*, and *ECE1*, the most well-known hypha-specific genes, were confirmed using qRT-PCR (Supplementary Fig. S2D–F). Furthermore, we observed that the hypha-specific genes were more highly expressed in *dset1* compared to WT, not only under normal conditions but also under hypha-inducing conditions (Supplementary Fig. S2C).

Consistent with the derepression of hypha-specific gene expression, we observed hyper-filamentous colonies of *dset1* under general serum-induced conditions (YPD supplemented with 10% FBS at 37°C), as well as in various carbon source media (Fig. 2C). Under glucose-growth conditions, *set1*-deleted strains exhibit yeast colonies that are not significantly different from the WT, displaying a smooth morphology. However, under other conditions, distinct hyphal forms are observed at the colony periphery and colonies exhibit a wrinkled and rough morphology associated with hypha, which is more pronounced in *dset1* than in WT [57, 58]. Furthermore, we observed the rapid morphogenesis of *dset1* at the indicated time point under low glucose YPD with serum and, surprisingly, *dset1* filamentous cells even without serum in the media (Fig. 2D). Thus, we observed abnormal expression of hypha-specific genes even under non-inducing conditions in the absence of Set1 and confirmed that this expression leads to faster hyphal formation in *dset1*.

### Upregulated genes in the absence of Set1 lack H3K4 methylation

**Figure 3.**
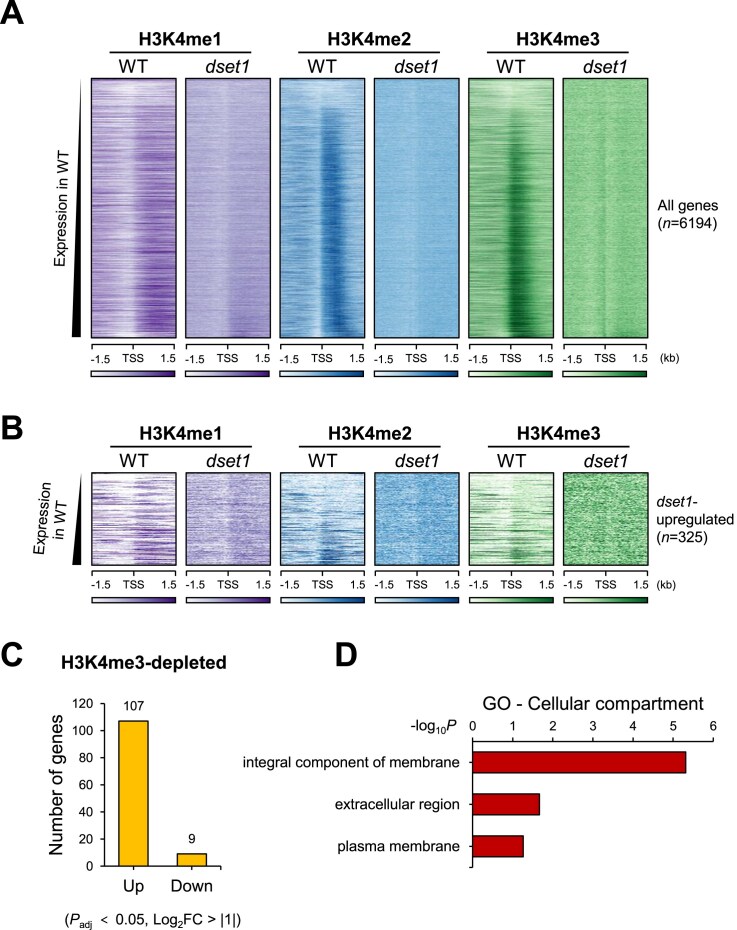
Upregulated genes in the absence of Set1 have the lack of H3K4 methylation. (**A**) Global H3K4 methylation ChIP-seq results correlated with the expression patterns as previously known. All 6194 genes were sorted based on their expression in WT. (**B**) Heatmap of H3K4 methylation enrichments in *dset1*-upregulated genes (log_2_FC > 1, *P*_adj_ < .05). The *dset1*-upregulated genes have low-level H3K4 methylation under normal condition. These *dset1*-upregulated genes were sorted according to H3K4me3 peaks in WT. All ChIP-seq data were sorted and visualized as a heatmap using EaSeq [56]. (**C**) H3K4me3-depleted gene expression was upregulated in *dset1*. H3K4me3 level was quantified using EaSeq. Most genes with low H3K4me3 had increased expression in deletion of Set1. (**D**) GO enrichment analysis revealed that the 107 *dset1*-upregulated genes with low H3K4me3 gene products were localized on cell surface.

Generally, H3K4me3 is localized in the 5′ region of actively transcribed genes [59]. As seen in the genome-wide heatmap, H3K4 methylation was proportional to the level of gene expression (Fig. 3A), which is consistent with previous studies. We observed that genes showing increased expression in the absence of Set1 generally lacked H3K4me1, -me2, and -me3 marks (Fig. 3B). We further confirmed H3 localization across these genes (Supplementary Fig. S3A), indicating that the depletion of H3K4 methylation aligns with the correlation between H3K4 methylation and gene expression levels. This correlation was evident because the genes that exhibited increased expression in the absence of Set1 were those with low expression levels in WT.

To identify whether H3K4me3 depletion is associated with increased expression in *dset1*, we quantified H3K4me3 peaks in the WT at the TSS (−100 to 300 bp) and categorized the expression patterns of the 500 genes with the lowest H3K4me3 abundance. Among the H3K4me3-depleted genes, 116 genes exhibited significant differential expression in *dset1* (*P*_adj_ < .05, log_2_FC > |1|). Notably, out of these, 107 genes were upregulated in *dset1*, while only 9 genes were downregulated (Fig. 3C). Moreover, 107 gene products exhibiting both depleted H3K4me3 levels and increased expression in *dset1* were also found to be located on the cell surface (Fig. 3D).

**Figure 4.**
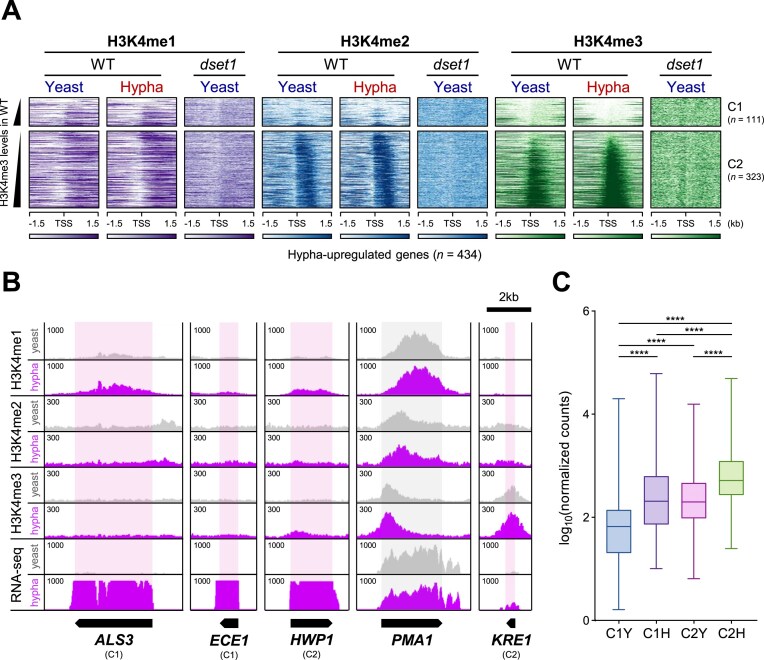
Hypha-upregulated genes have unusual H3K4 methylation patterns. (**A**) H3K4 methylation in yeast and hypha condition was unchanged in hypha-upregulated genes (log_2_FC > 1, *P*_adj_ < .05), despite the differential transcriptional levels in each condition. The 434 hypha-upregulated genes are categorized into two clusters based on the presence (cluster C2, *n* = 323) or absence (cluster C1, *n* = 111) of H3K4me3 in yeast condition. The heatmap is sorted by H3K4me3 levels in WT, and the color ramp represents density values ranging from 40 to 100. (**B**) Represented hypha-upregulated genes are nearly depleted of H3K4 methylation in both conditions, despite their transcription levels increasing by hundreds-fold during hyphal formation (see *ALS3*, *ECE1*, and *HWP1*), unlike *PMA1* gene. (**C**) The presence of H3K4me3 correlates with expression levels in the steady state without receiving any induction signals. The overall expression levels of each group in WT were depicted as log_10_-transformed DEseq2-normalized counts, illustrated in box plot. Statistical analysis was performed to compare expression levels between groups, and significant differences are denoted by *P < .0001. Non-significant comparisons are not explicitly marked in the figure.

To determine the role of H3K4 methylation-altered gene expression patterns, we used a transcriptional program during yeast-to-hypha morphogenesis in *C. albicans*. When *C. albicans* receives hyphal-inducing signals, it activates the expression of various genes necessary for hyphal formation. Thus, we attempted to determine the variation in the H3K4 methylation peak according to changes in gene expression status by comparing yeast and hyphal forms using ChIP-seq. However, no significant change was observed in H3K4 methylation among the 434 hypha-upregulated genes (log_2_FC > 1, *P*_adj_< .05) between the yeast and hyphal forms (Fig. 4A and Supplementary Fig. S4). In steady state, H3K4 methylation level significantly correlate with gene expression levels. However, under hypha conditions, despite a substantial increase in the expression levels of hypha-upregulated genes, H3K4 methylation does not increase and remains at levels similar to those observed under yeast conditions (Supplementary Fig. S4). In other words, H3K4 methylation did not seem to increase, despite a significant increase in the expression of hypha-upregulated genes in hyphal form. In particular, when we performed *k*-means clustering based on the presence or absence of H3K4me3 in yeast form (*k*= 2), we observed that, although the expression of hypha-upregulated genes increased in response to hypha-inducing signals, H3K4me3, which was absent in yeast form, did not appear in proportion to the expression levels (Fig. 4A).

As seen in the heatmap, H3K4 methylation in both yeast and hyphal forms remained unchanged, despite differences in the transcriptional levels between the two forms. The representative hypha-upregulated genes exhibited a near absence of H3K4 methylation in both forms, even though their transcript levels increased significantly during hyphal formation (see *ALS3*, *ECE1*, and *HWP1* in Fig. 4B). Despite *ALS3* and *ECE1* belonging to the C1 cluster, the *HWP1* gene is part of the C2 cluster, but it showed extremely low H3K4me3 in its TSS region. This is in contrast to the *PMA1* gene, which showed a high concentration of H3K4me3 at the TSS and a peak position change to the gene body for H3K4me2 and -me1 (Fig. 4B), as previously reported [60]. As shown in Fig. 4A, H3K4 methylation increased slightly in C2 of the hyphal form; therefore, we further examined the genes in C2, in particular *KRE1* gene, which showed the highest H3K4me3 enrichment. Although an increase in H3K4me3 was observed, H3K4me1 and -me2 did not exhibit typical enrichment patterns confirming that *KRE1* expression did not significantly increase in proportion to H3K4me3 level (Fig. 4B).

Supplementary Fig. S2B and C shows that H3 was well enriched in hypha-upregulated genes, indicating that the depletion of H3K4 methylation in these genes was not a result of histone eviction during transcription. Additionally, we investigated whether the depletion of H3K4me3 in hypha-upregulated genes was due to demethylation by Jhd2, an H3K4-specific demethylase. However, the knockout of *jhd2* showed no changes in the hyphal formation in *C. albicans* (Supplementary Fig. S5), indicating that H3K4me3 does not increase in hypha-upregulated genes.

We further explored the differences based on the presence or absence of H3K4me3 by comparing the expression levels in each group. We observed that genes with significant H3K4me3 presence in the yeast form (C2) were expressed at some level, even before receiving the hyphal induction signal. Thus, it seems that H3K4me3 is required for their steady state expression. Conversely, genes in C1 that completely lacked H3K4me3 exhibited minimal expression in yeast form but were strongly induced upon exposure to the hyphal signal (Fig. 4C). In other words, the presence of H3K4me3 correlated with basal expression levels in the steady state without any induction signal. However, there was no correlation when certain genes that were previously not expressed in response to any signal were newly induced. Overall, it was confirmed that even though H3K4 methylation is generally associated with active transcription and its peak correlates with expression level depending on the expression in the steady state, this correlation does not exist in hypha-upregulated genes, regardless of whether they are repressed or activated.

### H3K4 acetylation correlated with inducible gene expression

**Figure 5.**
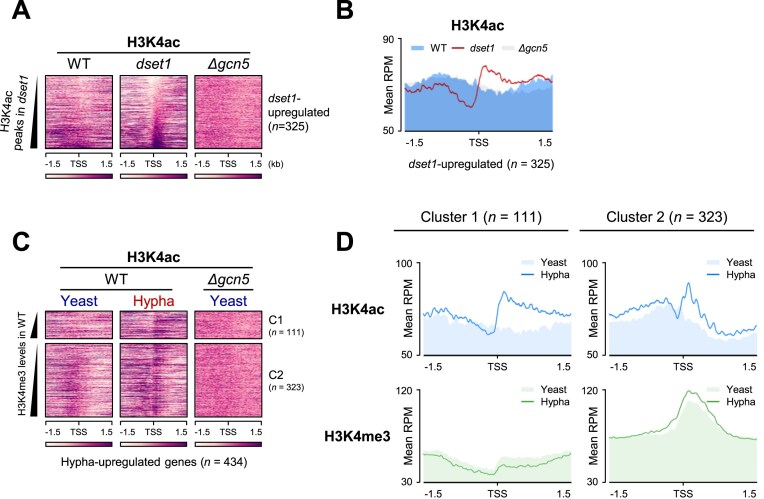
H3K4 acetylation is correlated with hypha-upregulated genes expression. (A, B) The lack of H3K4 methylation results in a significant enrichment of H3K4 acetylation in *dset1*-upregulated genes. Δ*gcn5* was used as a negative control for H3K4 acetylation. The 325 *dset1*-upregulated genes were sorted and depicted as a heatmap based on H3K4 acetylation peaks in *dset1* in panel (**A**). The average line plots of H3K4 acetylation ChIP-seq peaks are presented in panel (**B**). (**C**) H3K4 acetylation of hypha-upregulated genes increases in their transcriptionally activated state. The heatmap is sorted by H3K4me3 levels in WT, and the color ramp represents density values ranging from 40 to 100. (**D**) In metagene plot, it is confirmed that the H3K4 acetylation peak of hypha-upregulated genes, which did not exist in the yeast condition, occurs in the hyphal condition.

If H3K4 methylation were truly unrelated to the expression of genes activated by signals, then the absence of Set1 would not have led to any changes in gene expression. Although methylation is a major modification of H3K4, it has been reported that the H3K4 residue can undergo acetylation and the two modifications are mutually exclusive and are correlated with expression levels [14]. Furthermore, Set1 deletion caused a global increase in H3K4 acetylation in *Saccharomyces cerevisiae* [14]. To determine whether the acetyl group is localized at the unoccupied H3K4 residue of *dset1*-upregulated genes, we performed H3K4 acetylation ChIP-seq under the same conditions. As expected, we observed a significant increase in H3K4 acetylation peaks at the TSS of genes whose expression increased in the absence of Set1 and H3K4 methylation (Fig. 5A and B).

In addition, we found that H3K4 acetylation of hypha-upregulated genes increased under transcriptionally activated conditions (Fig. 5C). Unlike H3K4 methylation, which remained unchanged despite transcriptional state alterations, H3K4 acetylation increased with increased gene expression. The increase in H3K4 acetylation was more prominent in the H3K4me3-depleted hypha-upregulated genes (see C1 in Fig. 5C), indicating that low levels of H3K4me3 could effectively enhance H3K4 acetylation. Direct comparison using metagene plots confirmed that the H3K4 acetylation peak of hypha-upregulated genes, which did not exist in the yeast form, occurred in the hyphal form (Fig. 5D).

### H3K4 methylation and acetylation sites are interdependent

**Figure 6.**
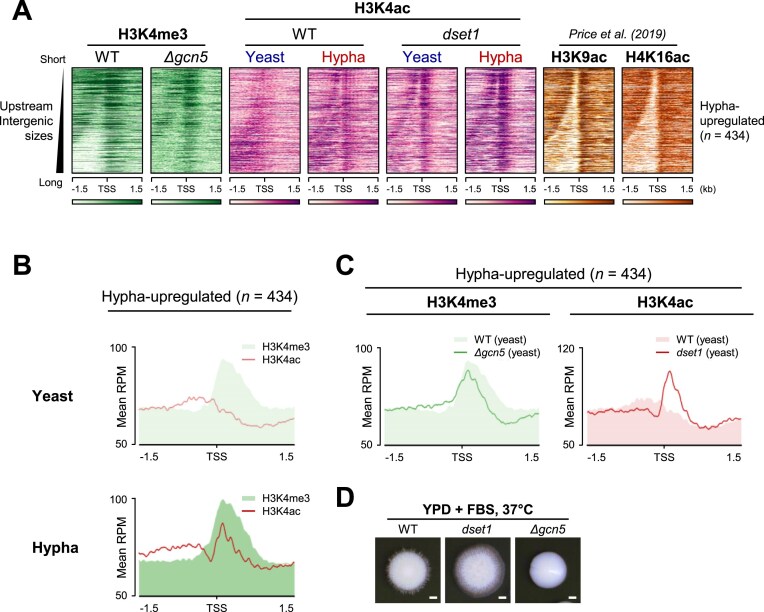
H3K4 methylation and acetylation location are affected by each other in distinct inducible genes. (**A**) H3K4 acetylation exhibits a distinct pattern unlike other major histone acetylation sites. H3K4 acetylation ChIP-seq data were sorted based on the length of upstream intergenic region. While most histone acetylations are concentrated at TSS like H3K4me3 [61], the H3K4 acetylation is enriched in the upstream intergenic regions from TSS since the H3K4me3 occupies the downstream of TSS. The H3K4 acetylation upstream peaks shift to TSS in the absence of Set1. (**B**) H3K4 acetylation and H3K4me3 have mutually exclusive positions for hypha-upregulated genes in yeast condition. In hyphal condition, however, H3K4 acetylation peaks are increased at the exact same position as H3K4me3, which is downstream of the typical H3K4 acetylation site. (**C**) Deletion of Gcn5 shifts the H3K4me3 peak upstream, creating a narrow peak on the TSS region, even in hypha-upregulated genes. (**D**) Δ*gcn5* failed to induce hyphal growth, in contrast to the hyper-filamentous form generated by *dset1* in serum inducing condition.

While investigating the localization of H3K4 modifications, we found that H3K4 acetylation exhibited a distinct pattern, unlike other major histone acetylation positions. Most histone acetylations, such as H3K9ac and H4K16ac, are localized at TSSs such as H3K4me3 [61]. However, we noticed that H3K4 acetylation was enriched in the intergenic regions upstream of the TSS, as H3K4me3 occupied the TSS (Fig. 6A). Given that H3K4 methylation primarily occurs within gene bodies, it appears that H3K4 acetylation is transferred from the TSS to intergenic regions. Intriguingly, in the absence of Set1, H3K4 acetylation was enriched in the TSS region, similar to other major histone acetylations (Fig. 6A). Moreover, upon induction of hyphal formation, we observed a shift in H3K4 acetylation from the upstream intergenic regions of the genes upregulated during hyphal formation to the TSS region, forming distinct peaks (Fig. 6A).

As H3K4 acetylation primarily occurs upstream of H3K4me3 [14], we compared the H3K4me3 and H3K4 acetylation peaks in the hypha-upregulated genes to determine the site of H3K4 acetylation. Figure 6B shows the mutually exclusive positions of H3K4 acetylation and H3K4me3 in hypha-upregulated genes in yeast form. However, H3K4 acetylation peaks increased at the exact same position as H3K4me3, downstream of the general H3K4 acetylation position, in the hyphal form (Fig. 6B). Overall, these results showed that H3K4 acetylation could replace H3K4me3 in inducible genes.

Our results suggest that H3K4 methylation regulates H3K4 acetylation by blocking H3K4 residues. Thus, to investigate the occurrence of reverse regulation, we examined the changes in H3K4 methylation in inducible genes under conditions where H3K4 acetylation does not occur. Unlike histone methylation, which is regulated by residue-specific writers, histone acetylation is affected by acetyltransferases with various substrate specificities. In *S. cerevisiae*, Gcn5 and Rtt109 were identified as complementary H3K4 acetyltransferases because H3K4 acetylation disappeared only in the *gcn5* and *rtt109* double deletion mutants [14]. In *C. albicans*, we confirmed that only Gcn5 was required for global H3K4 acetylation (Supplementary Fig. S6 and see Δ*gcn5* in Fig. 5A). To analyze the H3K4 methylation pattern in Δ*gcn5*, we performed H3K4me3 ChIP-seq in Δ*gcn5*. We found that deletion of Gcn5 shifted the H3K4me3 peak upstream, creating a narrow peak in the TSS region (Fig. 6C) even in hypha-upregulated genes. These results suggested that H3K4me3 alone in hypha-upregulated genes is insufficient for the expression of these inducible genes.

To further validate the observed increase in H3K4ac levels in *dset1* (Fig. 6C), we performed H3K4ac ChIP-seq with spike-in normalization using 5% *S. pombe* chromatin. Under yeast conditions, the absence of Set1 led to a substantial increase in H3K4ac levels at hypha-upregulated genes compared to WT (Supplementary Fig. S7). Notably, this increase was observed not only in the *dset1* strain used in this study but also in a Δ*set1* mutant, in which the entire *SET1* gene was deleted using the CRISPR–Cas9 system. These results confirm that the loss of Set1 consistently leads to elevated H3K4ac levels at hypha-specific genes, supporting the role of Set1-mediated H3K4 methylation in regulating H3K4 acetylation.

If H3K4 acetylation is indeed required for the expression of hypha-upregulated genes, the absence of *gcn5* cannot form hypha as opposed to that in *dset1*. A recent study revealed that Gcn5 is required for cell separation and normal morphogenesis in *C. albicans* [3, 44]. To confirm the relationship between Gcn5 and morphogenesis of *C. albicans*, we observed the hyphal formation in *gcn5* mutant strain (Δ*gcn5*) under serum-inducing condition. As expected, Δ*gcn5* did not form hypha, whereas *dset1* generated a hyper-filamentous form (Fig. 6D). As histone acetyltransferases are involved in the acetylation of several target residues, the inability of *C. albicans* to form hyphae in the absence of Gcn5 cannot be solely attributed to the role of H3K4 acetylation. Nevertheless, considering the cumulative results, it is evident that H3K4 acetylation plays a significant role in facilitating the expression of hypha-upregulated genes and hyphal formation and that Set1-mediated H3K4 methylation regulates H3K4 acetylation.

### Continuous expression of inducible genes is regulated by H3K4 methylation

**Figure 7.**
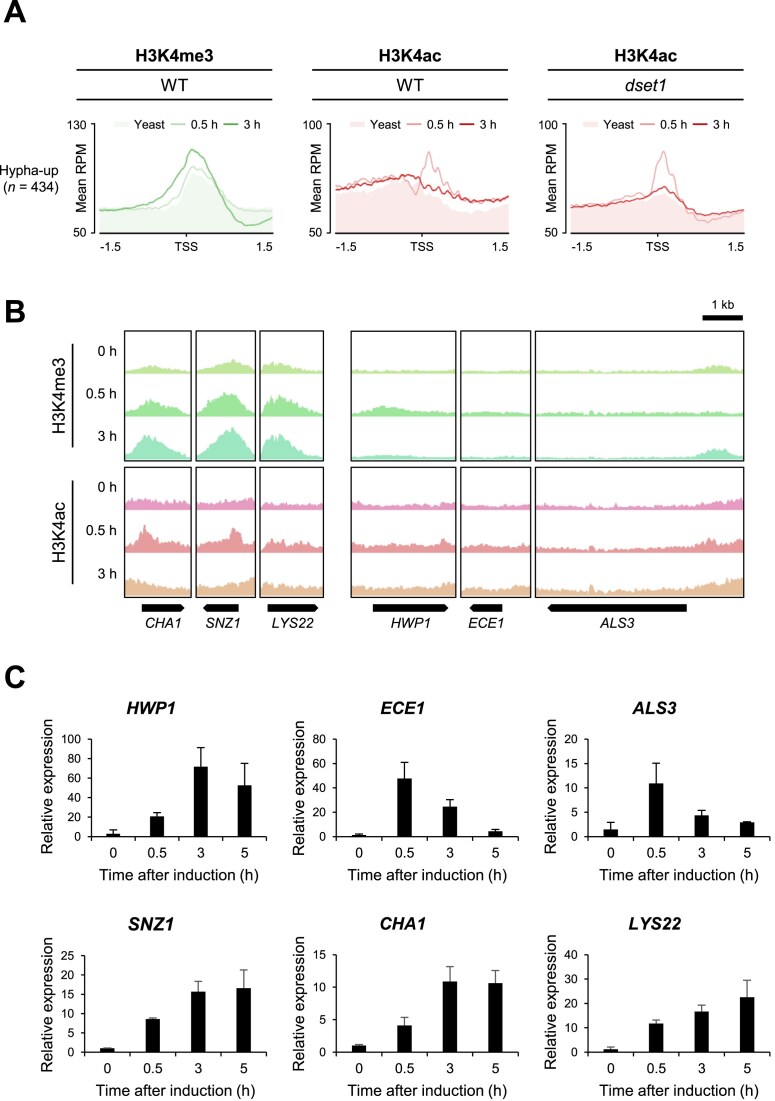
H3K4 methylation correlates with stable transcription during sustained induction. (**A**) Unusual H3K4me3 and H3K4 acetylation patterns were restored in long-term induction. (**B**) In hypha-upregulated genes, *CHA1*, *SNZ1*, and *LYS22* exhibited increased H3K4me3 levels during the 0–0.5 and 0.5–3 h of induction periods. However, *HWP1*, *ECE1*, and *ALS3* did not show H3K4me3 peaks. (**C**) *HWP1*, *ECE1*, and *ALS3* genes were early-induced hypha-upregulated genes and showed decreased expression levels during sustained induction. In contrast, *SNZ1*, *CHA1*, and *LYS22* genes exhibited constant increase in expression during hyphal induction. The expression value was normalized with *EFB1* housekeeping gene. Error bars indicate the standard deviation of three biological replicates.

In the initial stage of activation of inducible genes, we observed that these genes were regulated by distinct H3K4 modification patterns to achieve rapid and robust transcription. To determine whether the H3K4 modification pattern altered during sustained hyphal induction, we performed ChIP-seq after 3 h of induction and compared these H3K4 modification patterns with the data obtained after 30 min of induction. Interestingly, during the 3-h sustained induction of hypha-upregulated genes, we observed changes in the H3K4 modification pattern; H3K4me3, which did not increase at 30 min, increased, whereas the previously elevated H3K4 acetylation returned to a pattern resembling that of the yeast state (Fig. 7A). Furthermore, in *dset1*, when induction was prolonged, we observed a reduction in the increased H3K4 acetylation peak (Fig. 7A). Interestingly, when we examined the H3K4 methylation patterns of the 434 genes belonging to the hypha-upregulated gene group individually, not all genes exhibited the same pattern. Some genes, such as *HWP1*, *ECE1*, and *ALS3*, did not show an increase in H3K4me3 even after 3 h of induction, whereas others, such as *CHA1*, *SNZ1*, and *LYS22*, which are included in the C2 cluster, clearly exhibited elevated levels (Fig. 7B).

To investigate whether these differences were related to the expression patterns of each gene, we measured their expression levels over time during induction using qRT-PCR. The results showed that genes in which H3K4me3 increased after 3 h of induction were genes whose elevated expression levels, induced by hyphal induction, were either sustained or even further increased. In contrast, genes with increased H3K4me3 could not be observed even after 3 h of induction, as they exhibited rapid initial expression increases that decreased with increase in the duration of induction period (Fig. 7C). In repeated experiments with *dset1*, we observed that the expression levels of *HWP1* and *ECE1* were higher in *dset1* during the initial stages of induction. However, with prolonged induction, gene expression levels in *dset1* significantly decreased (Supplementary Fig. S8). This finding confirms the importance of H3K4me3 in maintaining steady expression. Thus, to examine whether genes in which H3K4me3 did not increase after 3 h of induction showed increased H3K4me3 levels at earlier time points, we examined their expression levels and H3K4me3 levels after 15 min of induction. We confirmed that *HWP1*, *ECE1*, and *ALS3* were early-induced hypha-specific genes, and an increase in H3K4 modification was observed at an early time point of induction. In contrast, *CHA1*, *SNZ1*, and *LYS22* were relatively late-induced and prolonged-induced hypha-upregulated genes, showing an early increase in H3K4 acetylation, followed by a later increase in H3K4me3 for prolonged expression (Supplementary Fig. S9A and B). In addition, we observed that H3K4me3 increased in *HWP1*, *ECE1*, and *ALS3* after 15 min of induction (Supplementary Fig. S9C). Overall, while there were differences in the timing and extent of induction when rapid induction of expression occurred, H3K4 acetylation was involved at an earlier time point and H3K4me3 was necessary to sustain this induced expression for each gene. Conversely, for genes whose expression no longer increased or decreased, it was evident that the increased H3K4 modification subsequently decreased. In conclusion, during the process by which the expression of previously silent inducible genes is promoted upon receiving signals, H3K4 methylation vacates its place to facilitate the rapid promotion of expression due to H3K4 acetylation and a transition from H3K4 acetylation to H3K4me3 occurs as the expression continues. Set1 precisely regulates the transcription of inducible genes, ensuring that it occurs at specific times at specific levels by precisely modulating H3K4 methylation and acetylation levels.

## Discussion

In this study, we elucidated the role of Set1 in the regulation of inducible gene transcription via H3K4me3 and subsequent H3K4 acetylation. Indeed, if Set1-mediated H3K4 methylation is important for triggering general transcriptional activation, yeast strains lacking *set1* should not survive owing to their inability to express most genes, including survival-related genes. However, the absence of Set1 in yeast results in few growth defects, and Set1 is not essential for survival [62, 63]. Unlike the downregulation of genes in *dset1*, the upregulation of genes in *dset1* could not be explained by the known roles of H3K4me3. Surprisingly, we found that H3K4 methylation did not increase in specific inducible genes, even under conditions where the expression of these genes was explosively induced. Furthermore, these genes were derepressed in the absence of *set1*, even under normal conditions. We discovered how and why the seemingly H3K4me3-independent genes exhibited dramatic expressional changes via acetylation of the methylation-depleted H3K4.

Generally, H3K4 acetylation is localized upstream of H3K4me3. Because methylation occurring at the same residue is much more enriched and significant, H3K4 acetylation has received relatively little attention. However, because histone acetylation can change the charge-based histone DNA interaction, we hypothesized that H3K4 acetylation substitutes for H3K4 methylation, at least in these inducible genes. As anticipated, our results showed that H3K4 acetylation, not H3K4me3, was increased in hypha-specific genes, especially under hypha-inducing conditions. As induction continues, H3K4me3 appears to be involved in sustaining stable transcription from the initial activation of inducible genes. Because transcriptional control between repression under normal conditions and explosive activation under hypha-inducing conditions of hypha-specific genes is important for *C. albicans*, hypha-specific genes seem to have a more specific regulatory mechanism than is generally known.

In the absence of *set1*, the hypha-specific genes lacked H3K4me1 to -me3, which resulted in increased H3K4 acetylation, promoting the derepression of these genes. Thus, Set1 regulates the transcriptional activation of inducible genes in response to signals and prevents aberrant transcription without signals (Supplementary Fig. S9D). The roles of Set1-mediated H3K4me3 in transcription have been debated for a long time. Recently, it has been widely accepted that H3K4me3 is more crucial for transcriptional consistency and memory rather than activation [22]. From this perspective, we elucidated that this is enabled by mild competition between methylation and acetylation at the same H3K4 residue, akin to musical chairs, using the *C. albicans* inducible gene turn-on system.

It is also possible that other histone acetylations are associated with Set1 and contribute to transcriptional regulation in the defective Set1 strain. In fission yeast, it has been shown that deletion of Set1 and the resulting loss of H3K4 methylation lead to a global decrease in various histone H3 acetylation marks, including H3K9ac and H3K14ac, particularly at constitutively expressed genes [64]. This study demonstrates a positive correlation between H3K4 methylation and histone acetylation, suggesting that both modifications are important for maintaining active transcription. While our study focused on the regulatory role of H3K4 acetylation in inducible genes that are upregulated in the absence of Set1, it remains possible that the altered transcriptional landscape observed in *C. albicans* lacking Set1 is due to combined changes in multiple histone acetylation marks. Future work will be necessary to investigate the broader contribution of other histone acetylations to Set1-dependent transcriptional regulation. In addition, while our study employed both a partial internal deletion (dset1) and a full gene deletion (Δ*set1*) to eliminate Set1 function, future efforts to generate catalytically inactive point mutants will be essential to distinguish the methyltransferase-dependent effects of Set1 from potential non-catalytic roles. Given the conservation of key catalytic residues across fungal species, such mutants could provide a more precise tool for dissecting the specific contribution of H3K4 methylation to gene regulation in *C. albicans*.

Each histone residue can undergo diverse modifications; therefore, the specific effects of each histone modification are well documented. However, there is still a limited understanding of the interplay of transcriptional regulation between modifications mediated by mutually exclusive writers competing for the same histone residue targets. Lysine residues are subjected to various modifications, including methylation, acetylation, ubiquitination, and SUMOylation. Among these, the roles of the different modifications occurring at the 9th and 27th lysine residues of H3, each involving methylation and acetylation, are well documented. H3 lysine 27 (H3K27) is a target of both acetyltransferase p300/CBP and Polycomb repressive complex 2. H3K27 methylation is characteristic of facultatively repressive chromatin and is conserved in many eukaryotes, excluding budding yeast or *Candida* species. In this inactive chromatin region, transcription occurs when the methyl group is removed from lysine 27 of H3 and the same residue undergoes acetylation. Therefore, H3K27me3 and H3K27 acetylation play opposing roles in transcription [65–67]. Similarly, methylation and acetylation of H3 lysine 9 (H3K9) have opposing roles, such as H3K27 modifications [68].

However, the precise interplay between the two modifications that affect transcription in the same direction, such as H3K4 methylation and acetylation, has not been well studied. Thus, the interrelationship between H3K4me3 and H3K4 acetylation in the positive regulation of transcription revealed in this study provides insights into lysine modifications occurring on residues known for their role in facilitating transcription through methylation, such as H3 lysine 36 and H3 lysine 79.

Since every eukaryotic organism has a chromatin structure to package DNA into its nucleus, histone modifications and histone modifiers that regulate chromatin structure have been actively studied using *S. cerevisiae*, the simplest yeast, as a model system. However, it is difficult to study the related physiology because of its limited phenotype. Unlike budding yeast, *C. albicans* can grow not only in a unicellular yeast form but also in a filamentous form. This morphological plasticity is very important for the virulence of *C. albicans*. Thus, *C. albicans* is another good model system to study how histone modifications and histone modifier enzymes regulate transcription and thereby identify their physiological roles.

## Supplementary Material

gkaf632_Supplemental_File

## Data Availability

Sequencing datasets generated and/or analyzed during this study have been deposited to the Gene Expression Omnibus Database (GEO) under accession numbers GSE246520, GSE246771, and GSE247148. All data generated or analyzed during this study are included in this published article and its supplementary information files.
