# Supplementary material for: Set1 is a critical transcriptional regulator in response to external signals in Candida albicans
Source: Nucleic Acids Res. 2025 Jul 8;53(13):gkaf632. doi: 10.1093/nar/gkaf632 (PMC12235511; doi:10.1093/nar/gkaf632)
Supplement: gkaf632_Supplemental_File [file gkaf632_supplemental_file.pdf]

Figure S1

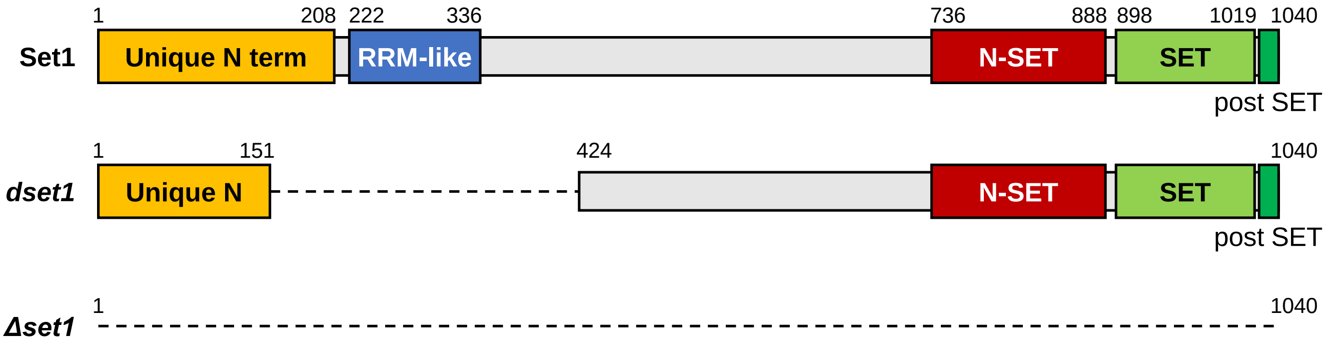

1
208
222
336
736
888
898
1019
1040
Set1
Unique N term
RRM-like
N-SET
SET
post SET
1
151
424
1040
dset1
Unique N
N-SET
SET
post SET
1
1040
Δset1

Figure S2

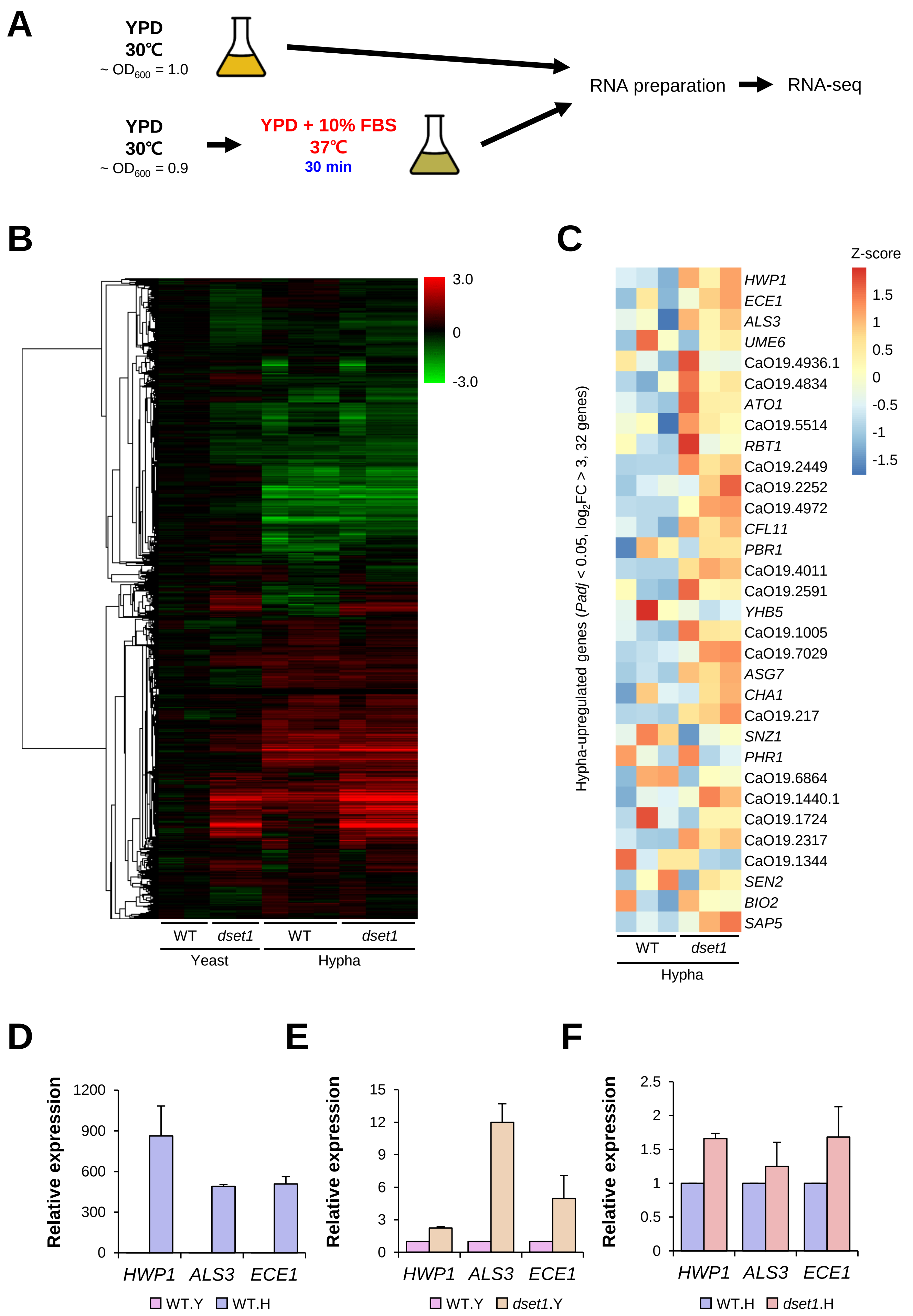

A
YPD
30℃
~ OD600 = 1.0
YPD
30℃
~ OD600 = 0.9
YPD + 10% FBS
37℃
30 min
RNA preparation
RNA-seq
B
3.0
0
-3.0
WT
dset1
WT
dset1
Yeast
Hypha
C
Z-score
1.5
1
0.5
0
-0.5
-1
-1.5
HWP1
ECE1
ALS3
UME6
CaO19.4936.1
CaO19.4834
ATO1
CaO19.5514
RBT1
CaO19.2449
CaO19.2252
CaO19.4972
CFL11
PBR1
CaO19.4011
CaO19.2591
YHB5
CaO19.1005
CaO19.7029
ASG7
CHA1
CaO19.217
SNZ1
PHR1
CaO19.6864
CaO19.1440.1
CaO19.1724
CaO19.2317
CaO19.1344
SEN2
BIO2
SAP5
Hypha-upregulated genes (Padj < 0.05, log2FC > 3, 32 genes)
WT
dset1
Hypha
D
Relative expression
1200
900
600
300
0
HWP1
ALS3
ECE1
WT.Y
WT.H
E
Relative expression
15
12
9
6
3
0
HWP1
ALS3
ECE1
WT.Y
dset1.Y
F
Relative expression
2.5
2
1.5
1
0.5
0
HWP1
ALS3
ECE1
WT.H
dset1.H

Figure S3

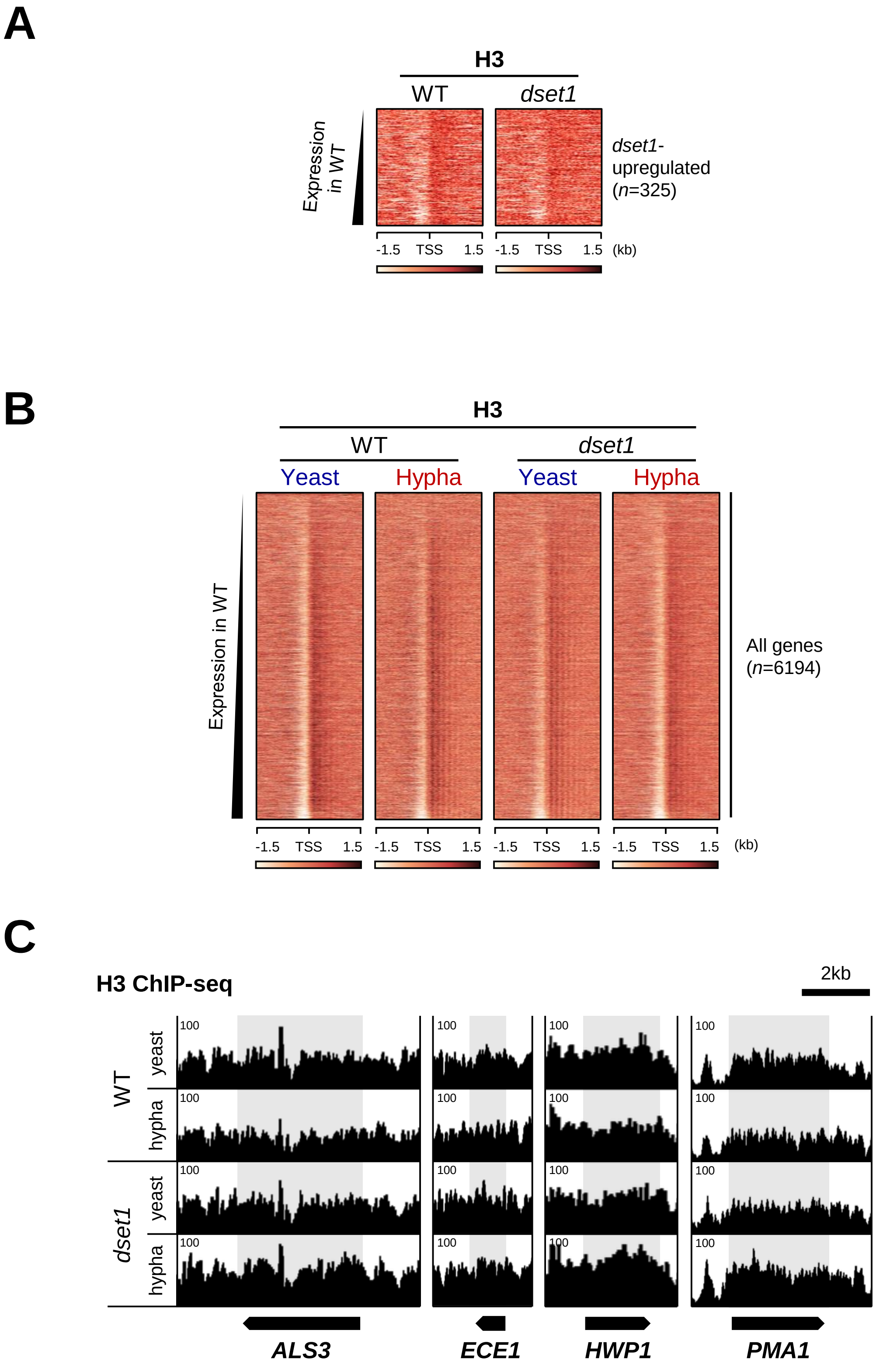

A
H3
WT
dset1
Expression in WT
dset1-upregulated (n=325)
-1.5 TSS 1.5 -1.5 TSS 1.5 (kb)
B
H3
WT
dset1
Yeast
Hypha
Yeast
Hypha
Expression in WT
All genes (n=6194)
-1.5 TSS 1.5 -1.5 TSS 1.5 -1.5 TSS 1.5 -1.5 TSS 1.5 (kb)
C
H3 ChIP-seq
2kb
WT
dset1
yeast
hypha
yeast
hypha
100
ALS3
ECE1
HWP1
PMA1

Figure S4

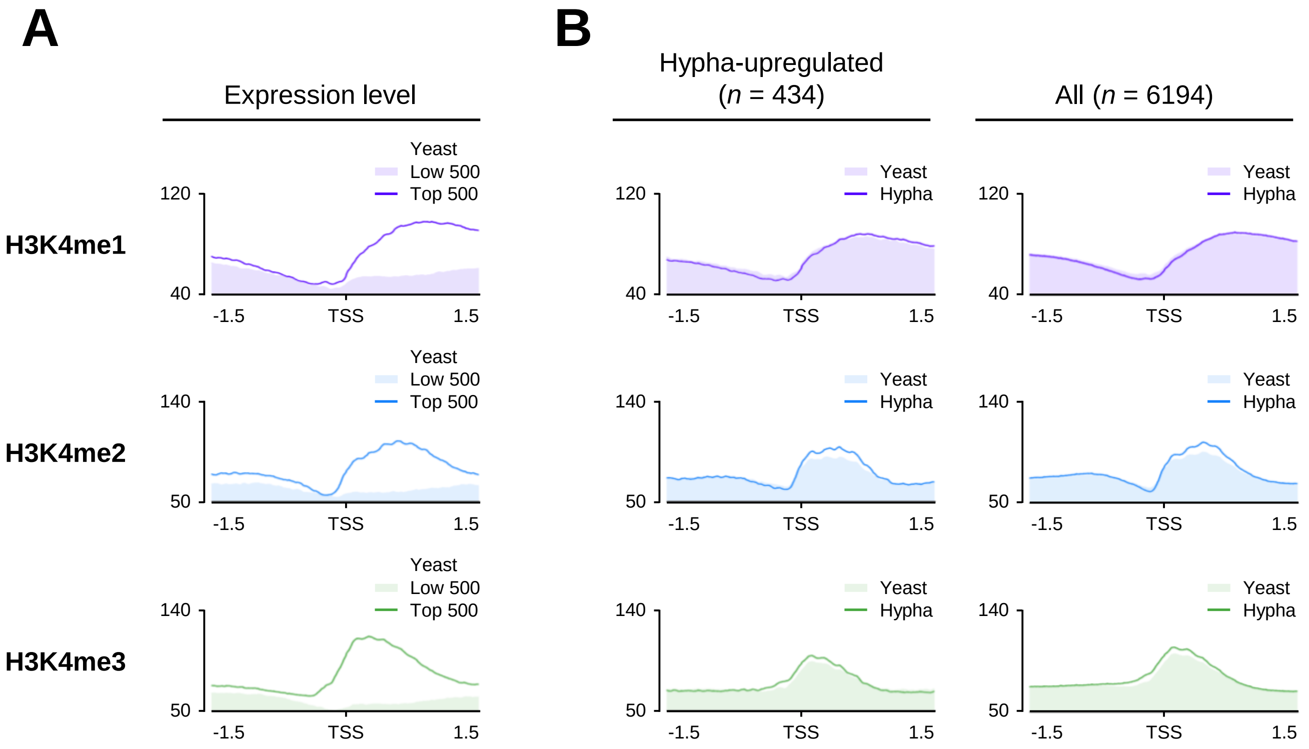

A
B
Expression level
Hypha-upregulated
(*n* = 434)
All (*n* = 6194)
H3K4me1
H3K4me2
H3K4me3
Yeast
Low 500
Top 500
Yeast
Hypha
120
40
140
50
-1.5
TSS
1.5

Figure S5

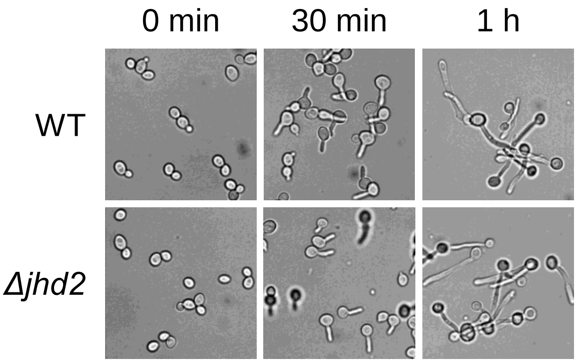

Figure S6

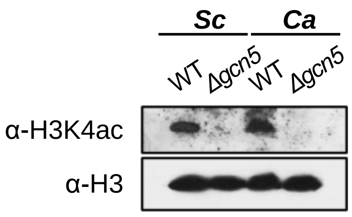

Figure S7

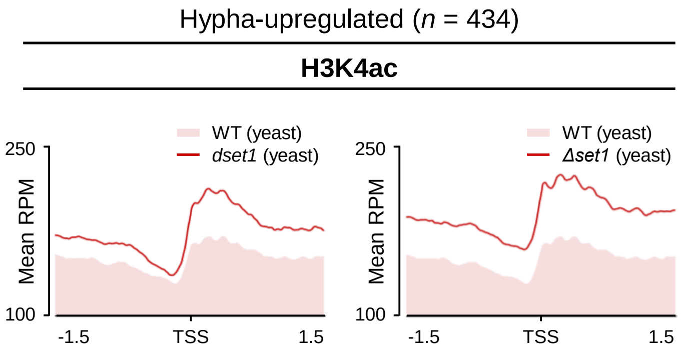
Hypha-upregulated (n = 434)
H3K4ac
WT (yeast)
dset1 (yeast)
250
100
Mean RPM
-1.5
TSS
1.5
WT (yeast)
Δset1 (yeast)
250
100
Mean RPM
-1.5
TSS
1.5

Figure S8

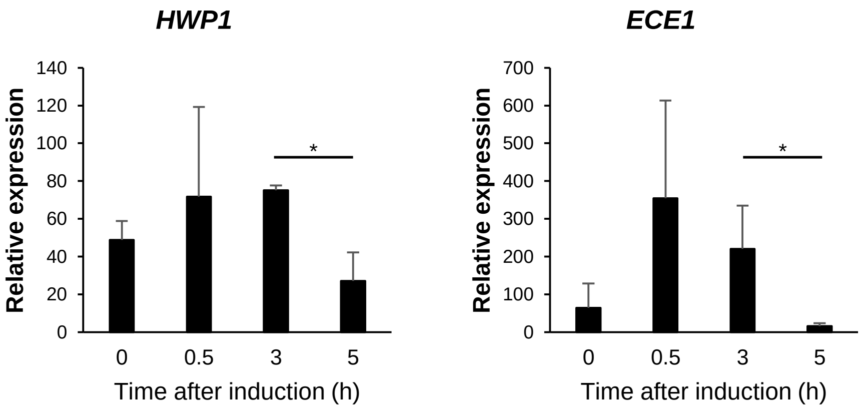

HWP1
ECE1
Relative expression
140
120
100
80
60
40
20
0
700
600
500
400
300
200
100
*
0
0.5
3
5
Time after induction (h)

Figure S9

A

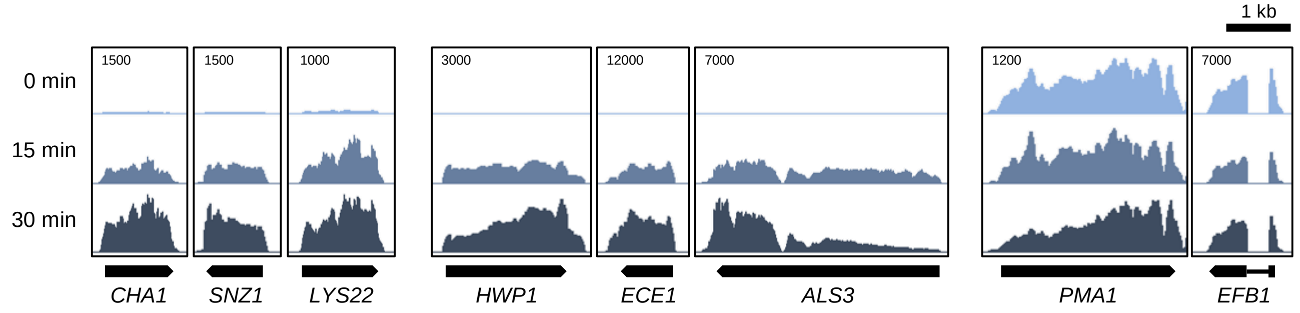

B

	DEseq2-normalized counts			log2foldchange	
	0 min	15 min	30 min	0 to 15	15 to 30
CHA1	398.7	2190.1	4502.3	2.5	1.0
SNZ1	1157.4	7348.5	12176.0	2.7	0.7
LYS22	615.1	3096.2	4188.1	2.3	0.4

	DEseq2-normalized counts			log2foldchange	
	0 min	15 min	30 min	0 to 15	15 to 30
HWP1	9.5	9693.1	13430.6	10.0	0.5
ECE1	51.5	16414.6	20717.1	8.3	0.3
ALS3	509.1	34145.5	29735.5	6.1	-0.2

C

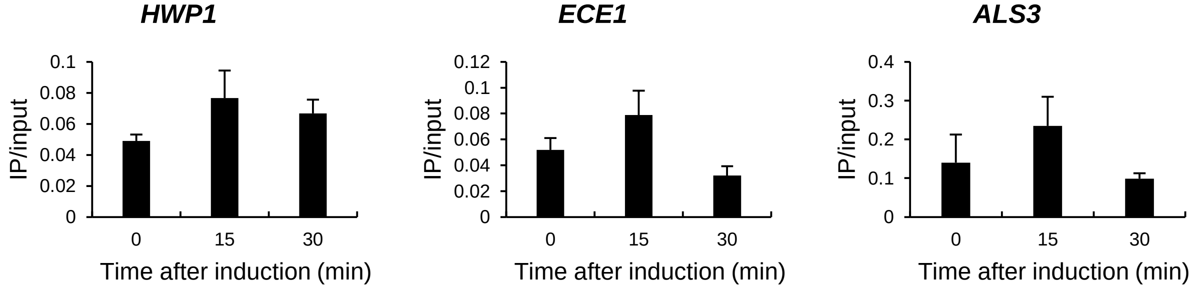

D

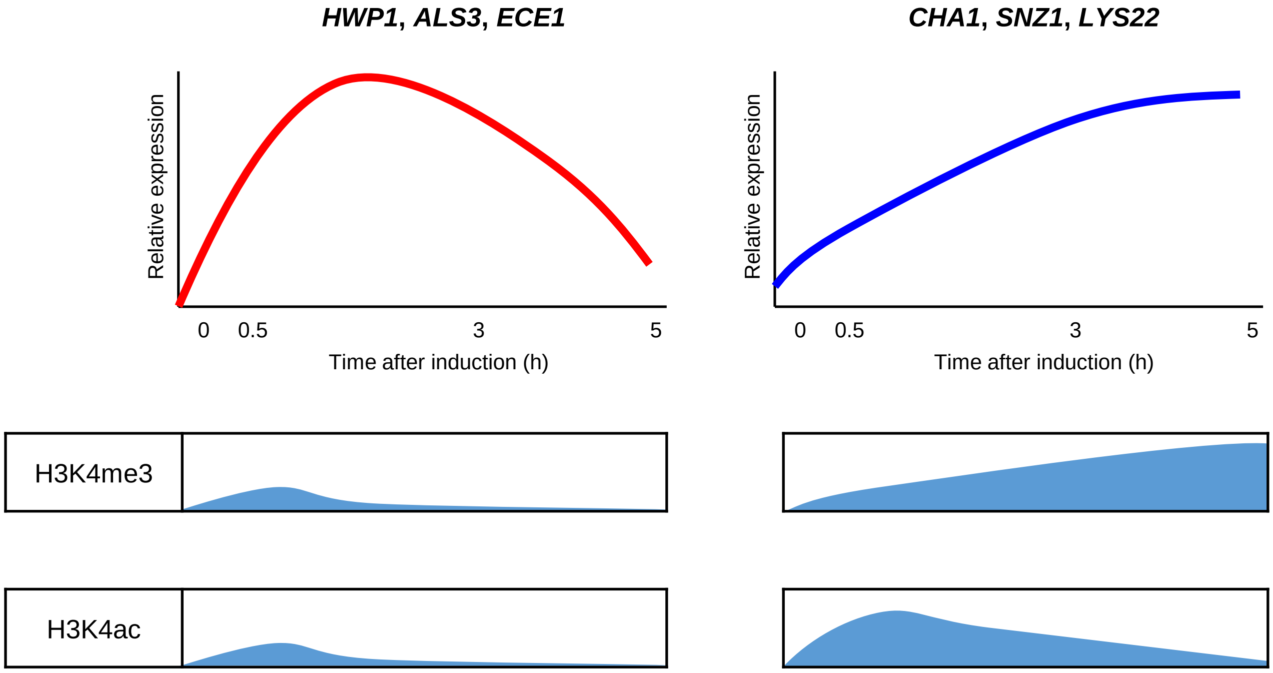

Figure S10

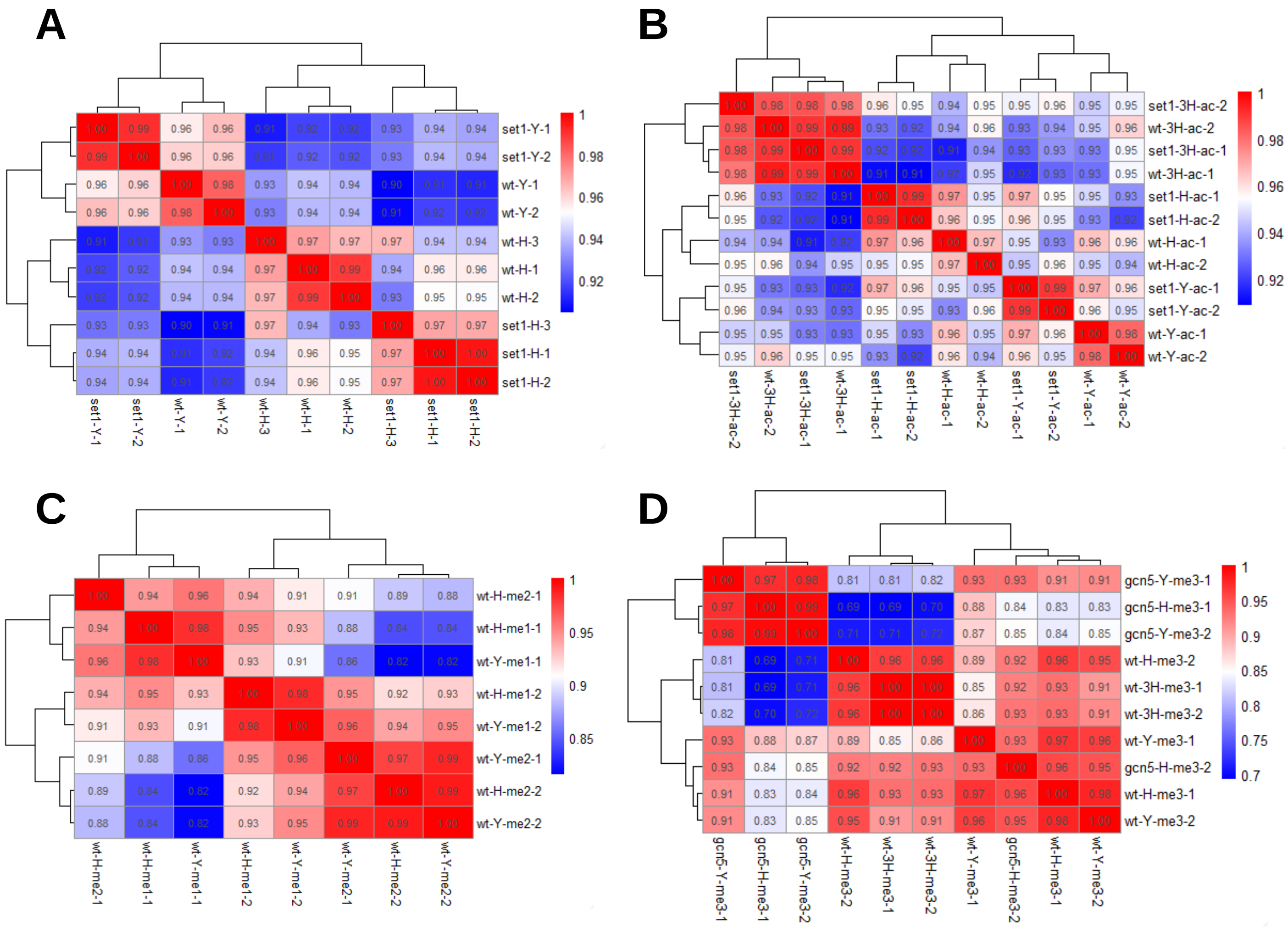
A
set1-Y-1
set1-Y-2
wt-Y-1
wt-Y-2
wt-H-3
wt-H-1
wt-H-2
set1-H-3
set1-H-1
set1-H-2
1
0.98
0.96
0.94
0.92
B
set1-3H-ac-2
wt-3H-ac-2
set1-3H-ac-1
wt-3H-ac-1
set1-H-ac-1
set1-H-ac-2
wt-H-ac-1
wt-H-ac-2
set1-Y-ac-1
set1-Y-ac-2
wt-Y-ac-1
wt-Y-ac-2
1
0.98
0.96
0.94
0.92
C
wt-H-me2-1
wt-H-me1-1
wt-Y-me1-1
wt-H-me1-2
wt-Y-me1-2
wt-Y-me2-1
wt-H-me2-2
wt-Y-me2-2
1
0.95
0.9
0.85
D
gcn5-Y-me3-1
gcn5-H-me3-1
gcn5-Y-me3-2
wt-H-me3-2
wt-3H-me3-1
wt-3H-me3-2
wt-Y-me3-1
gcn5-H-me3-2
wt-H-me3-1
wt-Y-me3-2
1
0.95
0.9
0.85
0.8
0.75
0.7

Supplementary Table 1. Primers used in this study.

Name	Primer sequences	Usage
F-HWP1-qRT	5'-CAGTTGCTCCAGGTACTGAAT-3'	RT-qPCR
R-HWP1-qRT	5'-GGAGCAATTGGTGAGGTTTC-3'	RT-qPCR
F-ALS3-qRT	5'-GCAATCCAATTCTGATACCCAAC-3'	RT-qPCR
R-ALS3-qRT	5'-ACCAGATCCGTCAAATGTAGTAAG-3'	RT-qPCR
F-ECE1-qRT	5'-GCTTTGAAAGACAGTCAACCAG-3'	RT-qPCR
R-ECE1-qRT	5'-TCTGAAACAATTTGAGCAGCAT-3'	RT-qPCR
F-SNZ1-qRT	5'-CAATTGGGATGTGATGGTGTTT-3'	RT-qPCR
R-SNZ1-qRT	5'-GTAGTGAGTGGTAGCGTTGAC-3'	RT-qPCR
F-CHA1-qRT	5'-GCAGCAATGGCAGTTGTTTAT-3'	RT-qPCR
R-CHA1-qRT	5'-AGTACATGAACCGCCACATAC-3'	RT-qPCR
F-LYS22-qRT	5'-CCATCTTGGCCAATCCATCA-3'	RT-qPCR
R-LYS22-qRT	5'-GGCATTCCAACCAGTCAATCTA-3'	RT-qPCR
F-HWP1	5'-CTCAACAATATCAAACACAACAGGA-3'	ChIP-qPCR
R-HWP1	5'-CGATAGCAATAAGTTGAGCAGTTG-3'	ChIP-qPCR
F-ALS3	5'-CTCCCTTGAATTGAGGTCTGATAG-3'	ChIP-qPCR
R-ALS3	5'-GCAGTCGCAACCGACAAATA-3'	ChIP-qPCR
F-ECE1	5'-TCTCTACAACAAACAACTTTCC-3'	ChIP-qPCR
R-ECE1	5'-TGGCAGCTTGAGAAGATAAA-3'	ChIP-qPCR
F-HMBS	5'- TTCTGTGCAGTGGACTTGAG-3'	ChIP-qPCR
R-HMBS	5'- GCTGAGCTCTGCGTCAC-3'	ChIP-qPCR
F-iNOS	5'-GTGTCACTGGTTTGAAACTTCTC-3'	ChIP-qPCR
R-iNOS	5'-TGGAGTGAACAAGACCCAAG-3'	ChIP-qPCR

Supplementary figure 1. Domain structure of Set1 mutants used in this study.
Schematic diagram of the *C. albicans* Set1 protein, showing conserved domains and the deletion boundaries of the two mutant alleles used. Conserved domains, including the RNA recognition motif (RRM)-like, N-SET, SET, and post SET domains, are indicated.

Supplementary figure 2. Set1 deletion causes signal-induced genes expression.
A, Cell preparation process for RNA-seq. For hyphal formation, cells grown under yeast conditions were induced at OD_{600} 0.9 by adding serum and incubating at 37°C.
B, Heatmap of total RNA expression in yeast or hyphal inducing condition. Raw RNA-seq data were normalized by library size using DESeq2 and transformed into a $\log_2$ scale. The fold changes toward WT under normal condition values were calculated and clustered using Cluster 3.0 and visualized using Java Treeview.
C, Hypha-specific genes were more expressed in *dset1* strain also in hyphal conditions. The upregulated 32 genes more than 8-fold change in hyphal conditions compared to the normal conditions of WT were classified as hypha-specific genes. Heatmap was visualized the same as Figure 2A.
D-F, The expression patterns of hypha-specific genes. Subsequent qRT-PCR was performed for *HWP1*, *ALS3*, and *ECE1* genes in the same conditions. The expression levels of each indicated strain were normalized with WT of normal conditions (D-E) or hyphal-inducing condition (F). Error bars indicate the SD of three biological replicates.

Supplementary figure 3. H3 is well enriched in the *dset1*-upregulated genes.
A, H3 was enriched in *dset1*-upregulated genes.
B, Global H3 ChIP-seq results were sorted based on their expression in WT.
C, H3 was well localized in represented hypha-specific genes.

Supplementary figure 4. H3K4 methylation is unrelated to inducible gene expression.
A, Metagene plots showing H3K4 methylation levels for the top 500 and low 500 genes based on expression levels under steady state yeast conditions. Low-expressing genes lack H3K4 methylation, while highly expressing genes exhibit high levels of H3K4 methylation.
B, Metagene plots comparing H3K4 methylation levels between yeast and hypha conditions for hypha-upregulated genes (n=434) and all genes (n=6194). Despite significant changes in expression levels for hypha-upregulated genes, their H3K4 methylation patterns remain similar

to those of all genes, indicating no correlation between H3K4 methylation and inducible gene expression changes.

Supplementary figure 5. Hyphal formation of the *jhd2*-deleted strain.

Hyphal differentiation was observed at the indicated time points after cells were transferred into fresh YPD supplemented with 10% FBS and incubated at 37°C. Deletion of *jhd2* did not impact on the morphogenesis of *C. albicans*.

Supplementary figure 6. Gcn5 was the sole acetyltransferase for histone H3K4.

Western blot analysis of H3K4 acetylation in *S. cerevisiae* (*Sc*) and *C. albicans* (*Ca*). FM391 is WT control for *S. cerevisiae Δgcn5*. Histone H3 was used as a loading control.

Supplementary figure 7. H3K4ac was highly enriched hypha-upregulated genes of *set1* mutant (related to Fig. 6C).

To further validate the findings in Figure 6C, we performed H3K4ac ChIP-seq with spike-in normalization using 5% *S. pombe* chromatin. Under yeast conditions, *dset1* exhibited a global increase in H3K4ac peaks at hypha-upregulated genes (n = 434) compared to WT. This pattern was consistently observed not only in the *dset1* strain used in this study but also in a *Δset1* mutant, in which the entire *SET1* gene was deleted. These results confirm that the absence of Set1 leads to a substantial increase in H3K4ac at hypha-specific genes, independent of the specific set1 deletion strategy used.

Supplementary figure 8. H3K4me3 promotes to stable transcription during sustained induction (related to Fig. 7C).

Hypha-specific gene expression levels were analyzed in *dset1* over a prolonged induction period. Initially, *dset1* exhibited higher expression levels in *HWP1* and *ECE1*. However, with sustained induction, expression levels in *dset1* gradually declined. Error bars indicate the SD of three biological replicates. Significant differences ($p < 0.05$) are denoted by an asterisk (*) in the figure.

Supplementary figure 9. Hypha-upregulated genes exhibit two distinct patterns in terms of both expression and H3K4 modifications.

A-B, RNA-seq analysis of hypha-upregulated gene expression patterns overtime. Hypha-

upregulated genes were grouped based on their induction and expression patterns. *CHA1*, *SNZ1*, and *LYS22* exhibited a moderate increase in expression levels overtime, whereas genes *HWP1*, *EFG1*, and *ALS3* showed rapid induction at the initial stage but displayed a declining rate of increase as time progressed.

C, *HWP1*, *EFG1*, and *ALS3* genes exhibiting a rapid and robust increase in expression levels followed by a decrease showed a faster occurrence and subsequent decline of H3K4me3 at earlier time points. Error bars indicate the SD of three biological replicates.

D, Hypha-upregulated genes can be classified into two groups based on their H3K4 modification and expression patterns. Cluster 1 initially lacks H3K4 modifications under yeast conditions but rapidly undergoes H3K4 acetylation and H3K4me3 upon hyphal induction, leading to a robust increase in expression at the early stage, followed by a subsequent decrease. In contrast, cluster 2 exhibits slight H3K4me3 even under yeast conditions, maintaining moderate expression levels. Upon hyphal induction, H3K4 acetylation occurs, resulting in a gradual increase in expression. As acetylation decreases and H3K4me3 levels rise, the expression remains stably increased over time.

Supplementary figure 10. Reproducibility analysis of RNA-seq and ChIP-seq data.

A, Pearson correlation heatmap of RNA-seq replicates, demonstrating high reproducibility across biological replicates.

B-D, Pearson correlation heatmap of H3K4ac (**B**), H3K4me1 and -me2 (**C**), and H3K4me3 (**D**) ChIP-seq replicates, confirming consistency between replicates.
